# Draft Genomes of Two Fusobacterium simiae Strains

**DOI:** 10.1128/mra.01255-22

**Published:** 2023-03-09

**Authors:** Malak Zoaiter, Pierre-Edouard Fournier, Linda Houhamdi

**Affiliations:** a IHU-Méditerranée-Infection, Marseille, France; b Aix-Marseille Univ, IRD, AP-HM, SSA, VITROME, Marseille, France; University of Maryland School of Medicine

## Abstract

Here, we reported two draft genomes of Fusobacterium simiae: strain DSM 19848, initially isolated from monkey dental plaque, and its close relative strain Marseille-Q7035, cultivated from a human intra-abdominal abscess puncture fluid. Their genome sizes are 2.4 Mb and 2.5Mb, respectively. Their G+C contents were 27.1% and 27.2%, respectively.

## ANNOUNCEMENT

Fusobacterium simiae, a Gram-negative bacterium belonging to the *Fusobacteriaceae* family, was described in 1982 (1). The type strain was first isolated from dental plaque of a monkey (Macaca arctoides) (2). As no genome sequence was available in GenBank, we purchased the type strain DSM 19848 from Deutsche Sammlung von Mikroorganismen (DSM) und Zellkulturen GmbH. Strain Marseille-Q7035, a closely related strain, was isolated from a 68-year-old woman’s intra-abdominal abscess on 5 January 2022, following appendectomy, in Marseille, France. The abscess was punctured, and 8 mL of the purulent fluid was placed in a hemoculture bottle. Growth occurred after 22.5 h of incubation at 37°C. The patient gave informed consent for this study according to the Declaration of Helsinki. Whole-genome sequencing was performed for the two strains to be compared. Both grew optimally at 37°C under anaerobic conditions on 5% sheep blood-enriched Columbia agar (bioMérieux, Marcy l’Etoile, France) after 24 h of incubation. Detailed culture conditions are provided in Table 1.

**TABLE 1:** Culture conditions of the two announced Fusobacterium simiae strains on 5% sheep blood-enriched Columbia agar: strain CSUR Q7035, cultivated from a human intra-abdominal abscess, and strain DSM 19848, initially isolated from monkey dental plaque^*a*^

Strain	Temp (°C)	Growth under condition for time (h)					
		Microaerophilic			Anaerobic		
		24	48	72	24	48	72
CSUR Q7035	RT	X	X	X	X	X	X
	28	X	√	√	X	√	√
	37	√	√	√	√	√	√

CSUR Q7974	RT	X	√	√	X	√	√
	28	√	√	√	√	√	√
	37	√	√	√	√	√	√

a RT, room temperature (20°C); X, no growth; √, positive growth. No bacterial growth was observed either in an aerobic atmosphere, or at 45°C and 56°C regardless of the tested atmosphere.

Genomic DNA was extracted on an EZ1 BioRobot (Qiagen, Hilden, Germany) using the EZ1 DNA tissue kit. A single colony of each strain was treated mechanically and chemically using glass beads and lysozyme, respectively, before DNA extraction. DNA was quantified using a Qubit assay (ThermoFisher Scientific). Sequencing was performed on a MiSeq sequencer (Illumina, San Diego, CA, USA) using a Nextera XT DNA sample prep kit (Illumina) and the mate pair strategy (3). Tagmentation was achieved using the Nextera XT transposome, which added index adapter sequences at the ends of fragmented DNA, allowing PCR amplification. The latter was performed with 12 limited cycles and introduced dual-index barcodes. Libraries were purified using AMPure XP beads (Beckman Coulter, Fullerton, CA, USA) and normalized using a bead-based procedure. Standardized libraries were combined, denatured, diluted, and pooled for sequencing. A single run was performed for cluster generation and paired-end sequencing in 2 by 250 bp with the MiSeq reagent kit (V2, 500 cycles).

Data of 6.2526 Gb for strain DSM 19848 were obtained from a 640-k/mm^2^ cluster density with 97.1% of clusters passing quality control filters. The run index representation was 5.78%. A total of 1,674,862 reads were produced by Illumina sequencing. For strain Marseille-Q7035, 9.287 Gb of data was obtained from a 1,577-k/mm^2^ cluster density with 63.5% of clusters passing quality control filters. The index representation was 9.05%. A total of 1,469,874 reads was obtained. The forward and reverse reads of both strains were quality checked using FastQC version 0.11.9 (4). They were trimmed on the Galaxy Europe server using Trimmomatic tool version 0.38.1 (5). They were assembled using SPAdes version 3.14.0 (6). The genomes were annotated using PGAP version 6.4 (7). All software programs were used with default parameters. Strain Marseille-Q7035 had a genome size of 2,430,878 bp with 27.2% G+C content. Strain DSM 19848 had a genome size of 2,481,730 bp with 27.1% G+C content. The genome-based comparison methods confirmed that strain Marseille-Q7035 was affiliated with the *F. simiae* species. Strain DSM 19848 was deposited in the Collection de Souches de l’Unité des Rickettsies as CSUR Q7974, and strain Marseille-Q7035 was deposited in the CSUR collection as CSUR Q7035 and in the Spanish Type Culture Collection as CECT 30592.

### Data availability.

Genome sequences of the two *F. simiae* strains Marseille-Q7035 and DSM 19848 were deposited in GenBank under accession numbers JAQQTB000000000 (https://www.ncbi.nlm.nih.gov/nuccore/JAQQTB000000000.1) and JAOXXL000000000 (https://www.ncbi.nlm.nih.gov/genome/?term=JAOXXL000000000), respectively, and the raw reads of each strain were deposited under accession numbers SRR23183347 (https://www.ncbi.nlm.nih.gov/sra/?term=SRR23183347) and SRR21745855 (https://www.ncbi.nlm.nih.gov/sra/?term=SRR21745855), respectively.
